# Outcomes of poor peripheral blood stem cell mobilizers with multiple myeloma at the first mobilization: A multicenter retrospective study in Japan

**DOI:** 10.1002/jha2.534

**Published:** 2022-07-21

**Authors:** Yurie Miyamoto‐Nagai, Naoya Mimura, Nobuhiro Tsukada, Nobuyuki Aotsuka, Masaki Ri, Yuna Katsuoka, Toshio Wakayama, Rikio Suzuki, Yoriko Harazaki, Morio Matsumoto, Kyoya Kumagai, Takaaki Miyake, Shuji Ozaki, Katsuhiro Shono, Hiroaki Tanaka, Arika Shimura, Yoshiaki Kuroda, Kazutaka Sunami, Kazuhito Suzuki, Takeshi Yamashita, Kazuyuki Shimizu, Hirokazu Murakami, Masahiro Abe, Chiaki Nakaseko, Emiko Sakaida

**Affiliations:** ^1^ Department of Hematology Chiba University Hospital Chiba Japan; ^2^ Department of Transfusion Medicine and Cell Therapy Chiba University Hospital Chiba Japan; ^3^ Division of Hematology Japanese Red Cross Medical Center Tokyo Japan; ^4^ Department of Hematology and Oncology Japanese Red Cross Narita Hospital Narita Japan; ^5^ Department of Hematology and Oncology Nagoya City University Graduate School of Medical Sciences Nagoya Japan; ^6^ Department of Hematology National Hospital Organization Sendai Medical Center Sendai Japan; ^7^ Department of Hematology and Oncology Shimane Prefectural Central Hospital Izumo Japan; ^8^ Department of Hematology and Oncology, Department of Medicine Tokai University School of Medicine Isehara Japan; ^9^ Department of Hematology Miyagi Cancer Center Natori Japan; ^10^ Department of Hematology National Hospital Organization Shibukawa Medical Center Shibukawa Japan; ^11^ Division of Hematology‐Oncology Chiba Cancer Center Chiba Japan; ^12^ Department of Oncology and Hematology Shimane University Hospital Izumo Japan; ^13^ Department of Hematology Tokushima Prefectural Central Hospital Tokushima Japan; ^14^ Department of Hematology Chiba Aoba Municipal Hospital Chiba Japan; ^15^ Department of Hematology Asahi General Hospital Asahi Japan; ^16^ Department of Hematology and Oncology, Graduate School of Medicine The University of Tokyo Tokyo Japan; ^17^ Department of Hematology National Hospital Organization Hiroshimanishi Medical Center Otake Japan; ^18^ Department of Hematology National Hospital Organization Okayama Medical Center Okayama Japan; ^19^ Department Clinical Oncology and Hematology The Jikei University Kashiwa Hospital Kashiwa Japan; ^20^ Department of Internal Medicine Keiju Kanazawa Hospital Ishikawa Japan; ^21^ Department of Hematology/Oncology Higashi Nagoya National Hospital Nagoya Japan; ^22^ Faculty of Medical Technology and Clinical Engineering Gunma University of Health and Welfare Maebashi Japan; ^23^ Department of Hematology, Endocrinology and Metabolism Tokushima University Graduate School Tokushima Japan; ^24^ Department of Hematology International University of Health and Welfare School of Medicine Narita Japan

**Keywords:** autologous stem cell transplantation, Multiple myeloma, peripheral blood stem cells, poor mobilizers

## Abstract

Autologous stem cell transplantation (ASCT) remains an important therapeutic strategy for multiple myeloma; however, a proportion of patients fail to mobilize a sufficient number of peripheral blood stem cells (PBSCs) to proceed to ASCT. In the present study, we aimed to clarify the characteristics and outcomes of poor mobilizers. Clinical data on poorly mobilized patients who underwent PBSC harvest for almost 10 years were retrospectively collected from 44 institutions in the Japanese Society of Myeloma (JSM). Poor mobilizers were defined as patients with less than 2 × 10^6^/kg of CD34^+^ cells harvested at the first mobilization. The proportion of poor mobilization was 15.1%. A sufficient dataset including overall survival (OS) was evaluable in 258 poor mobilizers. Overall, 92 out of 258 (35.7%) poor mobilizers did not subsequently undergo ASCT, mainly due to an insufficient number of PBSCs. Median OS from apheresis was longer for poor mobilizers who underwent ASCT than for those who did not (86.0 vs. 61.9 mon., *p* = 0.02). OS from the diagnosis of poor mobilizers who underwent ASCT in our cohort was similar to those who underwent ASCT in the JSM database (3y OS rate, 86.8% vs. 85.9%). In this cohort, one‐third of poor mobilizers who did not undergo ASCT had relatively poor survival. In contrast, the OS improved in poor mobilizers who underwent ASCT. However, the OS of extremely poor mobilizers was short irrespective of ASCT.

## 1 INTRODUCTION

High‐dose chemotherapy with autologous stem cell transplantation (ASCT) is an important treatment strategy for multiple myeloma (MM). Although the paradigm of MM treatment has markedly changed with the introduction of new drugs over the past decade, ASCT continues to have a significant impact on the outcomes of patients with MM [1] and is currently included as a standard treatment for eligible MM patients [2].

The collection of peripheral blood stem cells (PBSCs) must be performed prior to ASCT. Unfortunately, some patients fail to harvest a sufficient number of PBSCs for ASCT. The rate of poor mobilization varies depending on the mobilization strategy, but reportedly ranges between 5%–15% [3, 4, 5, 6, 7]. The poor mobilization of PBSCs sometimes leads to unsuccessful ASCT, which, in turn, results in dropouts from standard treatment. Therefore, many studies have focused on risk factors for poor mobilization or optimal strategies, such as the use of CXCR4 antagonists, to improve outcomes. Increasing age, low bone marrow cellularity, and prior chemotherapy or radiotherapy have been identified as risk factors, possibly triggered by a reduction in hematopoietic stem cell numbers in bone marrow [8].

In contrast, limited information is currently available on the prognosis of poor mobilizers. Based on the findings of their study on patients undergoing PBSC mobilization between 2001 and 2010, Moreb et al. concluded that poor mobilization was associated with poor outcomes in MM patients following ASCT [9]. However, this study did not focus on patients who had not undergone ASCT.

Therefore, the present study investigated the characteristics and outcomes of poor mobilizers at the first mobilization. We hypothesized that poor mobilizers had poorer survival than good mobilizers even in the era of novel treatment strategies.

## 2 PATIENTS AND METHODS

### 2.1 Study design and patients

This was a multicenter retrospective study conducted by 44 institutions in the Japanese Society of Myeloma (JSM). Clinical data on poorly mobilized patients with MM who underwent PBSC harvest (PBSCH) between April 2008 and September 2018 were collected. Based on the recommended number of CD34^+^ cells for ASCT [10], poor mobilizers in the present study were defined as those with less than 2 × 10^6^/kg of CD34^+^ cells harvested during the first mobilization, irrespective of the number of days of apheresis. Poor mobilizers included those who did not undergo apheresis because of the prediction of poor mobilization made by analysis of CD34 positive or hematopoietic progenitor cells in the peripheral blood according to each institution's criteria. The number of CD34^+^ cells collected was calculated as the sum of cells collected each day if PBSCH was performed across multiple days. The number of good mobilizers in the same period at the same institution was also examined to calculate the rate of poor mobilization. This study included patients treated in routine clinical practice in the participating institutions, and the decision of treatment strategy including ASCT was made based on each institution's criteria.

JSM previously conducted two retrospective cohort studies on MM. One study enrolled patients who were newly diagnosed between January 2001 and December 2012 [11], and the other included those who were newly diagnosed between January 2013 and December 2016 at institutions in JSM. We extracted data on MM patients who were newly diagnosed between April 2008 and December 2018 and younger than 70 years of age at the time of diagnosis from the database of these two studies, and defined it as the ‘JSM database cohort’. We then compared patient outcomes in the JSM database cohort with those of poorly mobilized patients diagnosed at the same period in the present study, which was defined as the ‘poor mobilization cohort’.

We retrieved the following information on poor mobilizers: age, sex, M protein type, clinical stages, cytogenetic abnormalities, treatments, treatment responses, procedures for mobilization, the number of PBSCs collected, subsequent ASCT, overall survival (OS), and cause of death. The diagnosis of MM was based on the International Myeloma Working Group criteria [12], and the clinical stage of MM was assessed by the Durie and Salmon (D and S) staging system [13] and International Staging System (ISS) [14]. Treatment options for each patient were selected by the attending physician. The response to treatment was evaluated by international uniform response criteria [15, 16].

The present study was approved by the Ethics Committee of Chiba University Graduate School of Medicine and the review committees of each institute.

### 2.2 Statistical analysis

Fisher's exact test was used to compare differences between categorical variables. Continuous or nominal values were analyzed by the Mann–Whitney *U* test. The Kaplan‐Meier method was performed to analyze OS, and differences between curves were examined using the Log‐rank test. The Cox proportional hazards model was applied to a multivariate analysis of independent predictors associated with survival. Statistical analyses were performed with R software version 3.3.2 and Graph Pad Prism version 8.3.0.

## 3 RESULTS

### 3.1 Characteristics of poor mobilizers

In the present study, 259 MM patients were extracted as poor mobilizers and 1714 patients were mobilized in the same period. The proportion of poor mobilization was 15.1%; it slightly increased after the introduction of lenalidomide for newly diagnosed patients (2016) and decreased after the approval of plerixafor (2017) in Japan (‐2015: 15.1%, 2016–2017: 18.1%, 2018: 5.1%) (Figure S1A). The incidence of poor mobilization varied between hospitals (median 14.8%, range 0%–50%) (Figure S1B).

Since one patient was excluded due to lack of sufficient dataset including OS, 258 poor mobilizers were subjected to analyses as the poor mobilizer group. The median follow‐up period from apheresis in these patients was 39.5 months (range 0.1–135.0 months). Baseline patient characteristics are summarized in Table 1. Median age at diagnosis was 61.8 years (range 29.9–77.3), and median age at the first course of apheresis was 62.4 years (range 30.3–77.6). The most frequent isotype of the M protein was IgG (51.9%), followed by the Bence‐Jones type (17.8%) and IgA (20.5%). Cases of plasmacytoma and non‐secretory myeloma were included as others (2.3%). In total, 180 patients (69.8%) were assigned to D&S stage III. ISS stages I, II, and III were distributed in 27.1, 31.0, and 36.4% of patients, respectively. Among 258 patients, the high‐risk chromosomal abnormalities t(4;14), t(14;16), and del(17p) were observed in 24 (9.3%), 6 (2.3%), and 18 (7.0%) patients, respectively.

**TABLE 1:** Baseline characteristics of poor mobilizers

	Overall	With autologous stem cell transplantation (ASCT)	Without ASCT	*p‐*Value
N	258	166	92	
Age at diagnosis, years (median [IQR])	61.75 (56.54, 64.95)	61.04 (54.76, 65.15)	61.92 (59.25, 64.76)	0.085
Age at apheresis, years (median [IQR])	62.42 (57.27, 65.52)	62.15 (55.75, 65.64)	62.65 (60.58, 65.39)	0.103
Male sex (%)	129 (50.0)	79 (47.6)	50 (54.3)	0.363
M protein (%)				0.588
IgG	134 (51.9)	81 (48.8)	53 (57.6)	
IgA	53 (20.5)	39 (23.5)	14 (15.2)	
BJP	46 (17.8)	28 (16.9)	18 (19.6)	
IgD	4 (1.6)	4 (2.4)	0 (0.0)	
IgG, BJP	10 (3.9)	6 (3.6)	4 (4.3)	
IgA, BJP	3 (1.2)	2 (1.2)	1 (1.1)	
IgG, IgA	1 (0.4)	1 (0.6)	0 (0.0)	
Other	6 (2.3)	4 (2.4)	2 (2.2)	
Unknown	1 (0.4)	1 (0.6)	0 (0.0)	
Free light chain (%)				0.405
κ	146 (56.6)	98 (59.0)	48 (52.2)	
λ	100 (38.8)	62 (37.3)	38 (41.3)	
Unknown	12 (4.7)	6 (3.6)	6 (6.5)	
Durie and Salmon stage (%)				0.397
I	25 (9.7)	15 (9.0)	10 (10.9)	
II	38 (14.7)	29 (17.5)	9 (9.8)	
III	180 (69.8)	112 (67.5)	68 (73.9)	
Unknown	15 (5.8)	10 (6.0)	5 (5.4)	
International Staging System (ISS) stage (%)				0.102
I	70 (27.1)	41 (24.7)	29 (31.5)	
II	80 (31.0)	50 (30.1)	30 (32.6)	
III	94 (36.4)	62 (37.3)	32 (34.8)	
Unknown	14 (5.4)	13 (7.8)	1 (1.1)	
Cytogenetic abnormalities (%)				
17p	18 (7.0)	12 (7.2)	6 (6.5)	1
t(4;14)	24 (9.3)	20 (12.0)	4 (4.3)	0.069
t(14;16)	6 (2.3)	3 (1.8)	3 (3.3)	0.756
Negative / Unknown	215 (83.3)	135 (81.3)	80 (87.0)	0.323
Number of treatment cycles before apheresis, months (median [IQR])	4.00 (4.00, 6.00)	4.00 (4.00, 6.00)	4.00 (3.00, 6.00)	0.961
Duration from diagnosis to apheresis, months (median [IQR])	6.33 (4.58, 9.26)	6.03 (4.45, 9.03)	6.60 (4.88, 10.83)	0.314
Treated with lenalidomide‐contained regimen (%)	89 (34.5)	61 (36.7)	28 (30.4)	0.376
Treatment response before apheresis (%)				0.001
VGPR≥	97 (37.6)	71 (42.8)	26 (28.3)	
VGPR<	153 (59.3)	94 (56.6)	59 (64.1)	
Unknown	8 (3.1)	1 (0.6)	7 (7.6)	
Mobilization regimen(%)				0.122
G‐CSF+Chemotherapy	162 (62.8)	97 (58.4)	65 (70.7)	
G‐CSF	94 (36.4)	68 (41.0)	26 (28.3)	
Unknown	2 (0.8)	1 (0.6)	1 (1.1)	
Harvested CD34+ cells at the first mobilization, 10^6^/kg (median [IQR])	0.90 [0.30, 1.53]	1.31 [0.60, 1.70]	0.32 [0.04, 0.80]	<0.001

IQR: interquartile range.

### 3.2 MM therapy before PBSC mobilization for poor mobilizers

Treatment regimens before PBSC mobilization are shown in Table S1. The median number of cycles of pre‐mobilization treatment was 4.0, and the median duration between diagnosis and PBSC collection was 6.33 months. Overall, 89 of 258 patients received at least one cycle of a lenalidomide‐containing regimen.

### 3.3 PBSC mobilization and collection for poor mobilizers

Regarding treatment responses before the first course of apheresis, 37.6% patients achieved very good partial response (VGPR) or greater (Table 1). At the first mobilization, the regimen of G‐CSF alone was used for 94 patients, G‐CSF following chemotherapy for 162, and G‐CSF with bortezomib for 8. Plerixafor was only used for seven patients in this cohort. The median number of days of apheresis was 2, and the median count of CD34^+^ cells harvested during the first mobilization was 0.90 × 10^6^/kg (range 0.00–1.98). Twenty‐eight patients did not receive apheresis because of the prediction of poor mobilization. Eighteen patients underwent the second mobilization, while two underwent the third mobilization. The median number of total CD34^+^ cells harvested through all mobilizations was 1.50 × 10^6^/kg (range 0.00–53.53), 55 of 258 patients (21.3%) achieved more than 2.0 × 10^6^/kg CD34^+^ cells in total, and 178 of 258 patients (69.0%) harvested more than 1.0 × 10^6^/kg CD34^+^ cells. Detailed information on mobilization and apheresis is shown in Table S2.

### 3.4 Subsequent ASCT for poor mobilizers

A total of 166 out of 258 poor mobilizers (64.3%) subsequently underwent ASCT. In contrast, 92 poor mobilizers did not undergo ASCT mainly due to an insufficient number of PBSCs (65 of 92, 70.7%). Reasons for avoiding ASCT, other than poor mobilization, included the progression of multiple myeloma or deterioration of general condition. The characteristics of poor mobilizers receiving ASCT are summarized in Supplemental Table 3. Median age at ASCT was 62.6 years (range 38.9–77.7). The median number of CD34^+^ cells infused in ASCT was 1.73 × 10^6^/kg (range 0.7 ‐ 8.4), and 44 of 166 patients (26.5%) underwent ASCT with more than 2.0 × 10^6^/kg CD34^+^ cells. All patients achieved engraftment; the median duration until the engraftment of neutrophils was 12 days, while that of platelets was 14 days. In total, 30.7% of patients received consolidation therapy after ASCT, while 38% received maintenance therapy. The treatment regimens of consolidation and maintenance therapies are shown in Supplemental Table 4.

### 3.5 Survival of poor mobilizers

**FIGURE 1:**
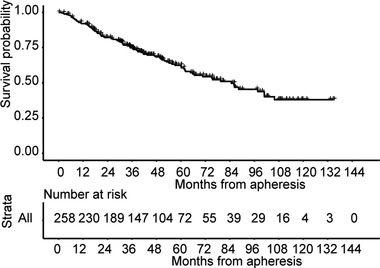
Survival of poor mobilizers. (A) Kaplan–Meier survival from apheresis of poor mobilizers

**FIGURE 2:**
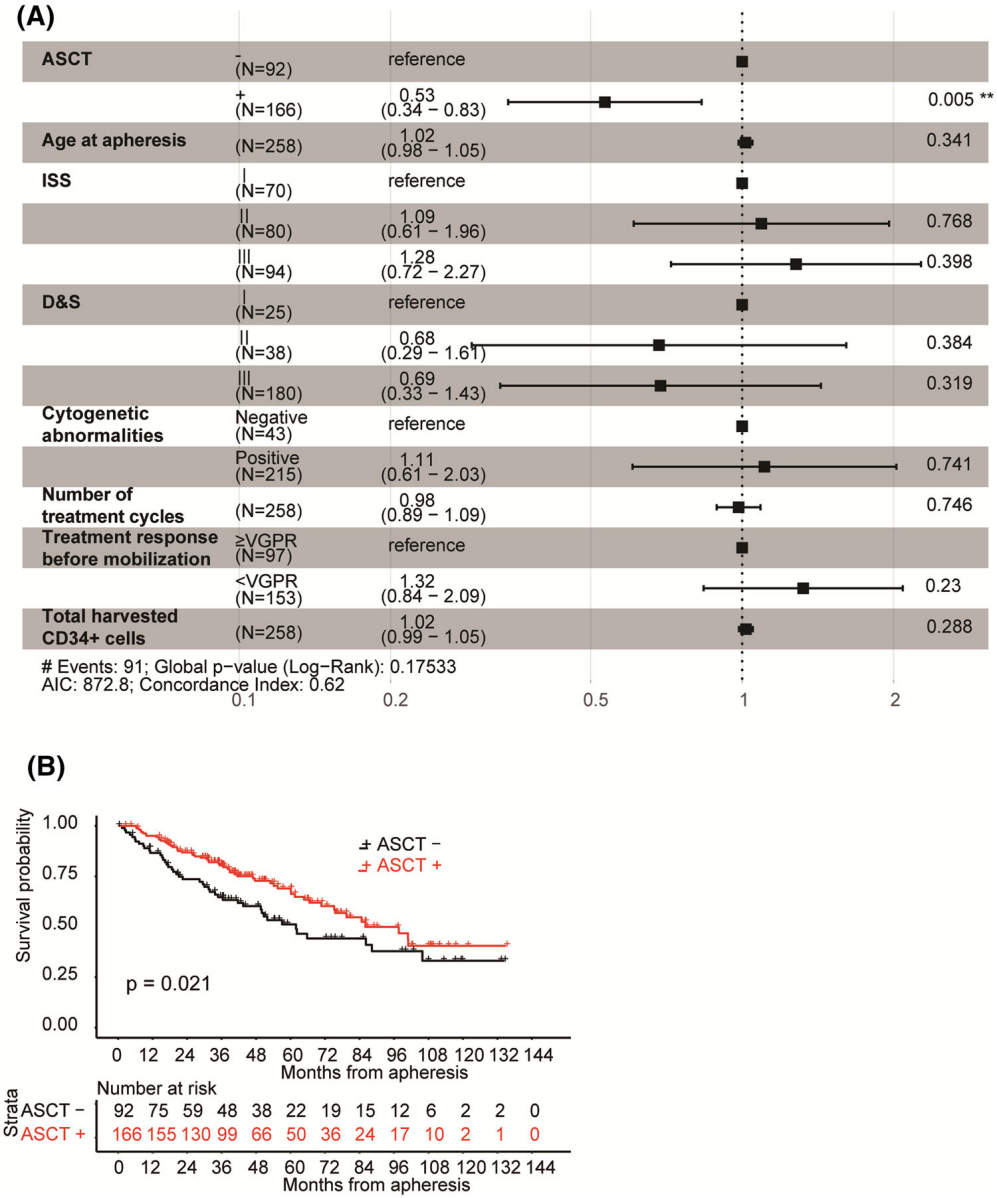
Survival was longer for poor mobilizers who underwent autologous stem cell transplantation (ASCT) than for those who did not. (A) A Cox proportional regression analysis of the overall survival of poor mobilizers. (B) Kaplan–Meier survival from apheresis of poor mobilizers according to the autologous stem cell transplantation (ASCT) status. The significance of differences between patients who underwent ASCT (ASCT +) and those who did not (ASCT ‐) was assessed using the Log‐rank test

The OS of poor mobilizers is shown in Figure 1. Median OS after the first course of apheresis was 84.7 months, and the 3‐year OS rate was 74.8% (Figure 1A). A Cox proportional regression analysis revealed that only the treatment of ASCT was independently associated with OS (hazard ratio, 0.53; 95% CI, 0.34–0.838, *p* = 0.005; Figure 2A).

We then separated poor mobilizers into two groups: those treated with or without ASCT. The baseline characteristics of the two groups are summarized in Table 1 and Tables S1 and S2. No significant differences were observed in the median follow‐up periods from apheresis between the two groups (with and without ASCT: 40.48 and 36.67 months, respectively). Age at diagnosis or at apheresis was higher in the group without ASCT than in that with ASCT (at diagnosis: 61.92 and 61.05 years; at apheresis: 62.65 and 62.15 years, respectively). More patients in the group with ASCT underwent second or third mobilization than those in the group without ASCT (47.6% and 21.7%, respectively). No significant differences were observed in D&S or ISS stages, treatment responses before apheresis, the number of treatment cycles before apheresis, or the duration from diagnosis to apheresis between the two groups. Median OS after the first course of apheresis was longer in the group with ASCT than in that without ASCT (86.0 vs. 61.9 months, *p* = 0.02; Figure 2B).

We then focused on the survival of poor mobilizers from the time of ASCT. The median follow‐up period from ASCT was 36.0 months (range 0.6–138.0), median OS from ASCT was 83.4 months, and the 3‐year OS rate was 76.8% (Figure S2A). Since 97 out of 166 patients relapsed after ASCT, median treatment‐free survival was 32.5 months (Figure S2B). The treatment response of ASCT was independently associated with OS after ASCT in these patients (hazard ratio, 2.12; 95% CI, 1.17–3.85, *p* = 0.013; Figure S2C).

### 3.6 Impact of extremely poor mobilization on survival

**FIGURE 3:**
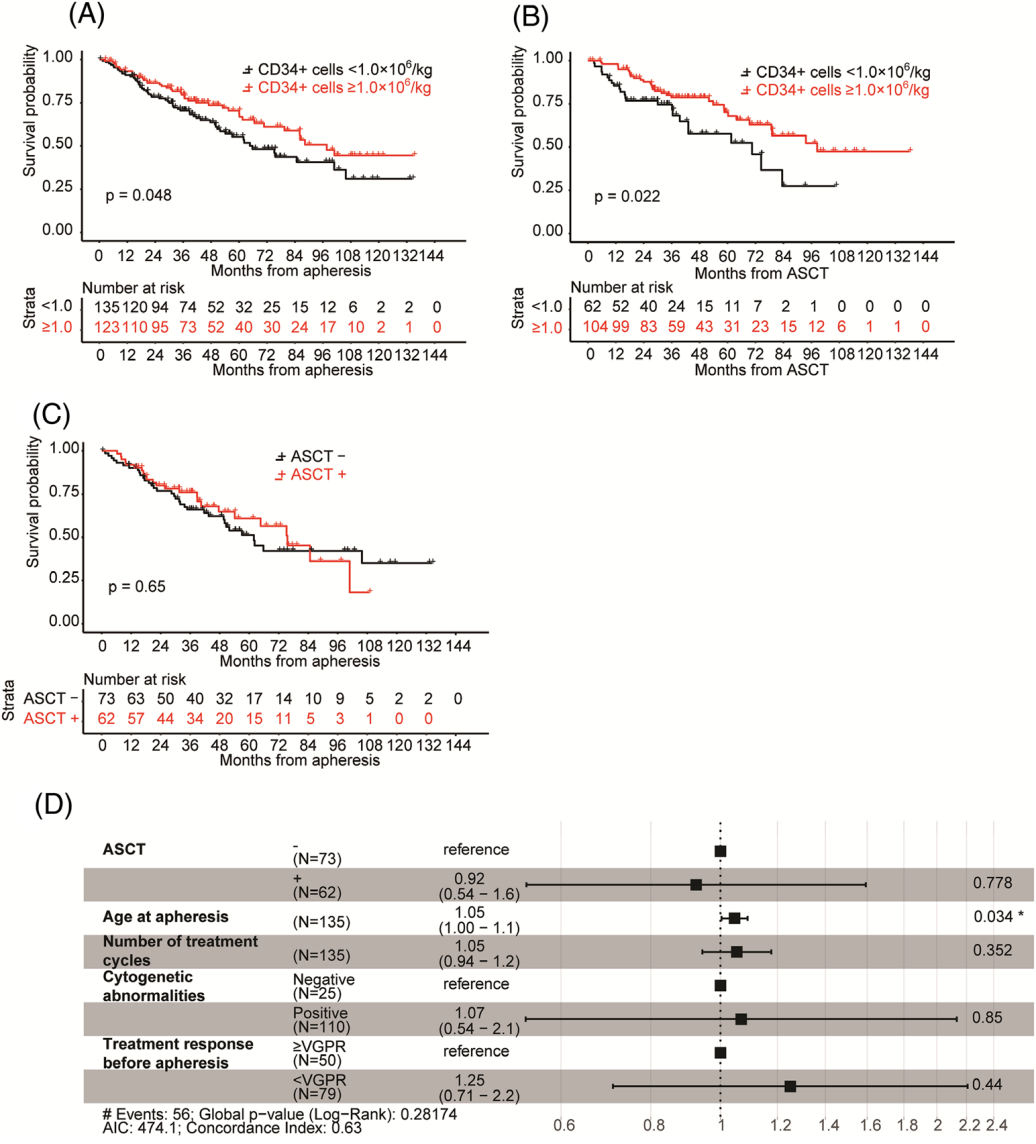
Extremely poor mobilizers had a poor prognosis. (A) Kaplan–Meier survival from apheresis of poor mobilizers according to the number of harvested CD34^+^ cells during the first mobilization. The significance of differences between these two groups was assessed by the Log‐rank test. (B) Kaplan–Meier survival from autologous stem cell transplantation (ASCT) of poor mobilizers who underwent ASCT according to the number of harvested CD34^+^ cells during the first mobilization. The significance of differences between these two groups was assessed by the Log‐rank test. (C) Kaplan–Meier survival from apheresis of extremely poor mobilizers (CD34^+^ cells < 1.0 × 10^6^/kg). The significance of differences between patients who underwent ASCT (ASCT +) and those who did not (ASCT ‐) was assessed using the Log‐rank test. (D) A Cox proportional regression analysis of the overall survival of extremely poor mobilizers

To analyze the survival of extremely poor mobilizers in the present cohort, we separated poor mobilizers using a cut‐off for the number of CD34^+^ cells of 1.0 × 10^6^/kg harvested during the first mobilization. A subset analysis of 135 extremely poor mobilizers (CD34^+^ cells < 1.0 × 10^6^/kg) was performed to assess the characteristics and survival of these patients in comparisons with 123 other poor mobilizers (CD34^+^ cells ≥1.0 × 10^6^/kg). The baseline characteristics of the two groups are summarized in Supplemental Table 5. Overall, 62 of 135 extremely poor mobilizers subsequently underwent ASCT, in contrast to 104 of 123 other poor mobilizers (45.9% vs. 84.6%, *p* = < 0.001). Although an insufficient number of stem cells was the main reason for avoiding ASCT for extremely poor mobilizers (59/73, 80.8%), it was not for other poor mobilizers (6/19, 31.6%). Figure 3A shows the survival curves of the two groups. Median OS after the first course of apheresis was shorter in extremely poor mobilizers than in other poor mobilizers (65.8 vs. 97.7 months, *p* = 0.048). Also, the outcome of extremely poor mobilizers who underwent ASCT was shorter than other poor mobilizers (median OS; 70.5 vs. 98.5 months, *p *= 0.022, Figure 3B). Engraftment of platelet in extremely poor mobilizers was significantly delayed compared to other poor mobilizers (Table S6). In extremely poor mobilizers, the OS of patients who underwent ASCT was not superior to those who did not (Figure 3C), and ASCT was not independently associated with survival (Figure 3D).

### 3.7 Comparison of the poor mobilization cohort with the JSM database cohort

The outcomes of 228 poor mobilizers in the poor mobilization cohort who were newly diagnosed between 2008/4 and 2018/3 at 70 years old or younger were analyzed and compared with the JSM database cohort. The baseline characteristics of the two cohorts are summarized in Table 2. Patients who underwent ASCT in the poor mobilization cohort were older than those in the JSM database cohort, whereas patients who did not undergo ASCT in the poor mobilization cohort were younger. No significant differences were observed in the D&S and ISS stages between these groups; however, cytogenetic high‐risk abnormalities were less frequently observed in the poor mobilization cohort than in the JSM database cohort based on available data. The median follow‐up period of patients who did not undergo ASCT from diagnosis was significantly longer in the poor mobilization cohort than in the JSM database cohort.

**TABLE 2:** Baseline characteristics of the poor mobilization cohort and Japanese Society of Myeloma (JSM) database cohort

	Poor mobilization cohort		JSM database cohort		
	With autologous stem cell transplantation (ASCT)	Without ASCT	With ASCT	Without ASCT	*p*‐Value
N	145	83	738	848	
Median follow‐up periods (median [IQR])	50.63 (35.43, 79.63)	44.47 (27.05, 69.55)	40.82 (26.73, 60.34)	29.25 (13.66, 50.68)	<0.001
Age at diagnosis (median [IQR])	61.00 (54.62, 64.72)	62.08 (59.91, 64.66)	58.84 (53.00, 63.00)	65.00 (60.00, 67.48)	<0.001
Age at apheresis (median [IQR])	50.63 (35.43, 79.63)	44.47 (27.05, 69.55)	40.82 (26.73, 60.34)	29.25 (13.66, 50.68)	<0.001
Male sex (%)	67 (46.2)	46 (55.4)	418 (56.6)	465 (54.8)	0.149
M protein (%)					<0.001
IgG	69 (47.6)	47 (56.6)	432 (58.5)	450 (53.1)	
IgA	35 (24.1)	14 (16.9)	130 (17.6)	157 (18.5)	
BJP	25 (17.2)	16 (19.3)	94 (12.7)	121 (14.3)	
IgD	2 (1.4)	0 (0.0)	23 (3.1)	14 (1.7)	
IgE	0 (0.0)	0 (0.0)	1 (0.1)	1 (0.1)	
IgM	0 (0.0)	0 (0.0)	2 (0.3)	5 (0.6)	
IgG, BJP	6 (4.1)	4 (4.8)	0 (0.0)	1 (0.1)	
IgA, BJP	2 (1.4)	1 (1.2)	0 (0.0)	0 (0.0)	
IgG, IgA	1 (0.7)	0 (0.0)	1 (0.1)	1 (0.1)	
Other	4 (2.8)	1 (1.2)	0 (0.0)	0 (0.0)	
Unknown	1 (0.7)	0 (0.0)	55 (7.5)	98 (11.6)	
Free light chain (%)					0.008
Κ	86 (59.3)	43 (51.8)	412 (55.8)	427 (50.4)	
Λ	55 (37.9)	35 (42.2)	271 (36.7)	327 (38.6)	
Unknown	4 (2.8)	5 (6.0)	55 (7.5)	94 (11.1)	
Durie and Salmon stage (%)					<0.001
I	13 (9.0)	7 (8.4)	72 (9.8)	91 (10.7)	
II	21 (14.5)	8 (9.6)	167 (22.6)	160 (18.9)	
III	101 (69.7)	64 (77.1)	416 (56.4)	429 (50.6)	
Unknown	10 (6.9)	4 (4.8)	83 (11.2)	168 (19.8)	
International Staging System (ISS) stage (%)					<0.001
I	37 (25.5)	26 (31.3)	254 (34.4)	244 (28.8)	
II	44 (30.3)	26 (31.3)	294 (39.8)	285 (33.6)	
III	56 (38.6)	30 (36.1)	175 (23.7)	279 (32.9)	
Unknown	8 (5.5)	1 (1.2)	15 (2.0)	40 (4.7)	
Cytogenetic abnormalities					
17p	10 (6.9)	6 (7.2)	125 (16.9)	141 (16.6)	0.002
t(4;14)	20 (13.8)	3 (3.6)	81 (11.0)	75 (8.8)	0.042
t(14;16)	3 (2.1)	3 (3.6)	22 (3.0)	18 (2.1)	0.638
Negative / Unknown	116 (80.0)	72 (86.7)	555 (75.2)	653 (77.0)	0.089

**FIGURE 4:**
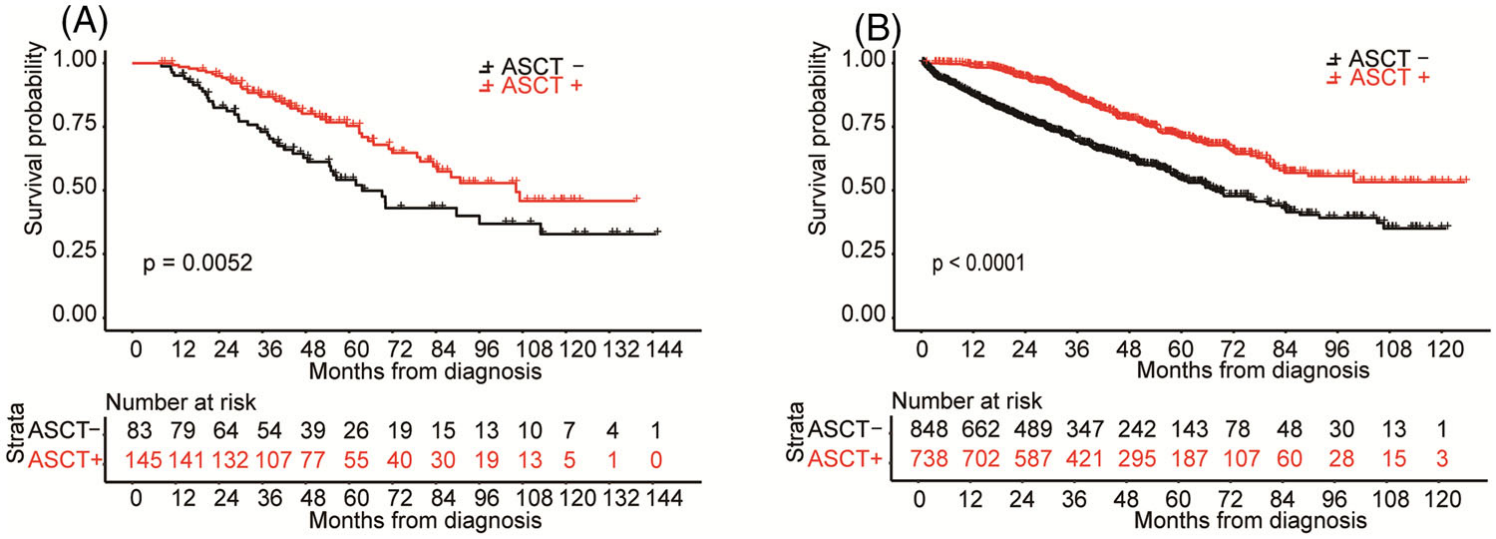
Overall survival (OS) from diagnosis of poor mobilizers who underwent autologous stem cell transplantation (ASCT) in our cohort was similar to that of those who underwent ASCT in the Japanese Society of Myeloma (JSM) database cohort. (A and B) Kaplan–Meier survival from diagnosis of poor mobilizers (A), the JSM database (B). The significance of differences between patients who underwent ASCT (ASCT +) and those who did not (ASCT ‐) was assessed using the Log‐rank test

In both cohorts, OS after diagnosis was longer in patients who underwent ASCT than in those who did not (Figure 4A,B) (poor mobilization cohort: *p *= 0.005, JSM database cohort: *p* < 0.001). OS from diagnosis was similar in poor mobilizers who underwent ASCT in our cohort and those who underwent ASCT in the JSM database cohort (3y OS rate, 86.8 vs. 85.9%).

## 4 DISCUSSION

In the present study, we performed a retrospective analysis of poor mobilizers, who were defined as MM patients with less than 2.0 × 10^6^/kg CD34^+^ cells harvested at the first mobilization, at 44 institutions in Japan for more than 10 years. We defined 15.1% of MM patients as poor mobilizers; however, 69.0% of poor mobilizers had more than 1.0 × 10^6^/kg CD34^+^ cells after multiple mobilizations, and up to two‐thirds of poor mobilizers subsequently underwent ASCT. In contrast, 92 poor mobilizers did not undergo ASCT mainly due to insufficient number of PBSCs. Poor mobilizers who did not undergo ASCT had a relatively poor prognosis; however, the survival of poor mobilizers who underwent ASCT was similar to that of newly diagnosed MM patients who underwent ASCT in the JSM database cohort.

Initial treatment strategies for MM were changed following the introduction of novel drugs, such as bortezomib and lenalidomide, which may affect the results of PBSCH. More importantly, the introduction of the CXCR4 inhibitor plerixafor had a major impact on the mobilization of stem cells in peripheral blood. A decrease was observed in the rate of poor mobilization after the approval of plerixafor in the present study, indicating that more MM patients were able to undergo ASCT.

Previous studies identified the following risk factors for poor mobilization: age, exposure to melphalan or lenalidomide, bone marrow infiltration, and mobilization regimens [17, 18, 19, 20]. Risk factors for poor mobilization, such as age and disease status, are partly shared with those of a poor prognosis in MM patients undergoing ASCT. Therefore, poor mobilizers had been considered to have a poor prognosis even after ASCT, as previously reported [9]. In contrast, the present study demonstrated that poor mobilizers who underwent ASCT did not have a poorer prognosis. This discrepancy may be attributed to improvements in post‐transplant treatment options for MM. Several consolidation and maintenance therapies using immunomodulatory drugs, proteasome inhibitors, and monoclonal antibodies have been developed, which improve the survival of poor mobilizers. Median OS of poor mobilizers from ASCT was 55.8 months in the study by Moreb et al [9], but that in the present study was 83.4 months. This difference can be explained not only by the different definitions of poor mobilizers, but also by the different periods of the two studies. On the other hand, the survival of poor mobilizers who did not undergo ASCT had been unknown. The present results revealed poor OS of poor mobilizers who did not undergo ASCT even by novel therapeutic options. Salvage therapies may not be effective for and may be tolerated less by poor mobilizers who did not undergo ASCT than by those undergoing ASCT; however, this was not verified in the present study.

We focused on the severity of poor mobilization and found that more than 80% of extremely poor mobilizers (CD34^+^ cells < 1.0 × 10^6^/kg) did not subsequently undergo ASCT because of an insufficient number of stem cells, which was associated with poorer survival than in other poor mobilizers. Furthermore, in extremely poor mobilizers, ASCT did not improve their survival. One possibility is tumor contamination in autologous grafts in this population, which reportedly contributes to poorer survival [21]. Another possibility is the poor quality of stem cells, which may be improved by the introduction of plerixafor [22]. The ratio of plerixafor introduction was only 8.9% (12/135) in extremely poor mobilizers in the present study; however, the use of plerixafor is currently increasing in Japan and may improve the survival of MM patients. Stem cells from poor mobilizers more frequently contained variants from clonal hematopoiesis of indeterminant potential (CHIP) than good mobilizers [23]. Therefore, CHIP may contribute to the poor quality of stem cells and affect the outcomes of ASCT. Moreover, patients with CHIP may have difficulties in post‐transplantation treatments with complications such as cytopenia.

## 5 CONCLUSION

In this cohort, one third of poor mobilizers at the first mobilization who did not subsequently undergo ASCT had relatively poor survival. In contrast, improved OS was observed in poor mobilizers who received ASCT. The OS of extremely poor mobilizers was poor irrespective of ASCT and needs to be improved.

## CONFLICT OF INTEREST

N. Tsukada received honoraria from Sanofi, Janssen Pharmaceutical and Takeda Pharmaceutical. R. Suzuki received honoraria from Janssen Pharmaceutical. M. Matsumoto received honoraria from Janssen Pharmaceutical and Sanofi. K. Sunami received research funding from Ono Pharmaceutical, MSD, Celgene, AbbVie, Takeda Pharmaceutical, Sanofi, Bristol Myers Squibb, Daiichi Sankyo, Janssen Pharmaceutical, Novartis, Alexion Pharmaceuticals, GlaxoSmithKline, and Astellas‐Amgen, and honoraria from Ono Pharmaceutical, Celgene, Takeda Pharmaceutical, Bristol Myers Squibb, Sanofi, and Janssen Pharmaceutical. K. Suzuki received honoraria from Takeda Pharmaceutical, Janssen Pharmaceutical, and Sanofi. M. Abe received research funding from Chugai Pharmaceutical, Sanofi, Pfizer, Kyowa Kirin, MSD, Astellas Pharmaceutical, Takeda Pharmaceutical, Teijin Pharma, and Ono Pharmaceutical, and honoraria from Daiichi Sankyo and Janssen Pharmaceutical. C. Nakaseko received honoraria from Fujimoto Pharmaceutical, Janssen Pharmaceutical, and Takeda Pharmaceutical. E. Sakaida received research funding from Chugai Pharmaceutical, Ono Pharmaceutical, and Kyowa Kirin, and honoraria from Novartis, Pfizer, and Bristol Myers Squibb. No other potential conflict of interest relevant to this article was reported.

## FUNDING INFORMATION

The authors received no specific funding for this work.

## AUTHOR CONTRIBUTIONS

Y. Miyamoto‐Nagai designed the research, collected, analyzed, interpreted data, and actively wrote the manuscript. N. Mimura (the corresponding author) conceived and designed the research, analyzed, interpreted data, and actively wrote the manuscript. N. Tsukada, N. Aotsuka, M. Ri, Y. Katsuoka, T. Wakayama, R. Suzuki, Y. Harazaki, M. Matsumoto, K. Kumagai, T. Miyake, K. Shono, H. Tanaka, A. Shimura, Y. Kuroda, K. Sunami, K. Suzuki, and T. Yamashita collected data. S. Ozaki collected, analyzed, and interpreted data. K. Shimizu, H. Murakami, M. Abe, C. Nakaseko, and E. Sakaida analyzed and interpreted data.

## Supporting information

Suporting information

## Data Availability

The data that support the findings of this study are available from the corresponding author upon reasonable request.
