# Supplementary material for: Outcomes of poor peripheral blood stem cell mobilizers with multiple myeloma at the first mobilization: A multicenter retrospective study in Japan
Source: EJHaem. 2022 Jul 21;3(3):838–48. doi: 10.1002/jha2.534 (PMC9422024; doi:10.1002/jha2.534)
Supplement: Supplementary file 1 — Suporting information [file JHA2-3-838-s001.pdf]

Outcomes of poor peripheral blood stem cell mobilizers with multiple myeloma at the first mobilization

Yurie Miyamoto-Nagai, Naoya Mimura, Nobuhiro Tsukada, Nobuyuki Aotsuka, Masaki Ri, Yuna Katsuoka, Toshio Wakayama, Rikio Suzuki, Yoriko Harazaki, Morio Matsumoto, Kyoya Kumagai, Takaaki Miyake, Shuji Ozaki, Katsuhiro Shono, Hiroaki Tanaka, Arika Shimura, Yoshiaki Kuroda, Kazutaka Sunami, Kazuhito Suzuki, Takeshi Yamashita, Kazuyuki Shimizu, Hirokazu Murakami, Masahiro Abe, Chiaki Nakaseko, and Emiko Sakaida

<u>Supporting Information:</u>

Supplemental Figures and legends 1-2

Supplemental Tables 1-6

Supplemental information

Supplemental Figure 1.

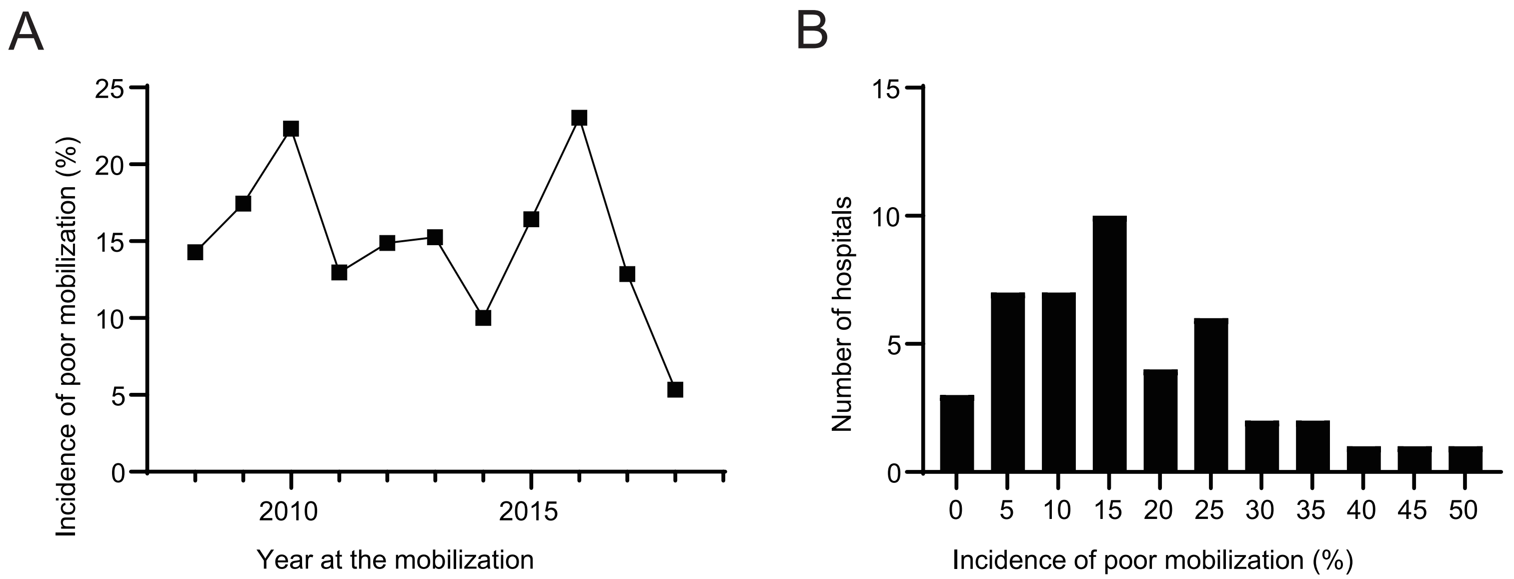

Supplemental Figure 1. Incidence of poor mobilization.

(A, B) Incidence of poor mobilization according to time periods (A) and hospitals (B).

Supplemental Figure 2.

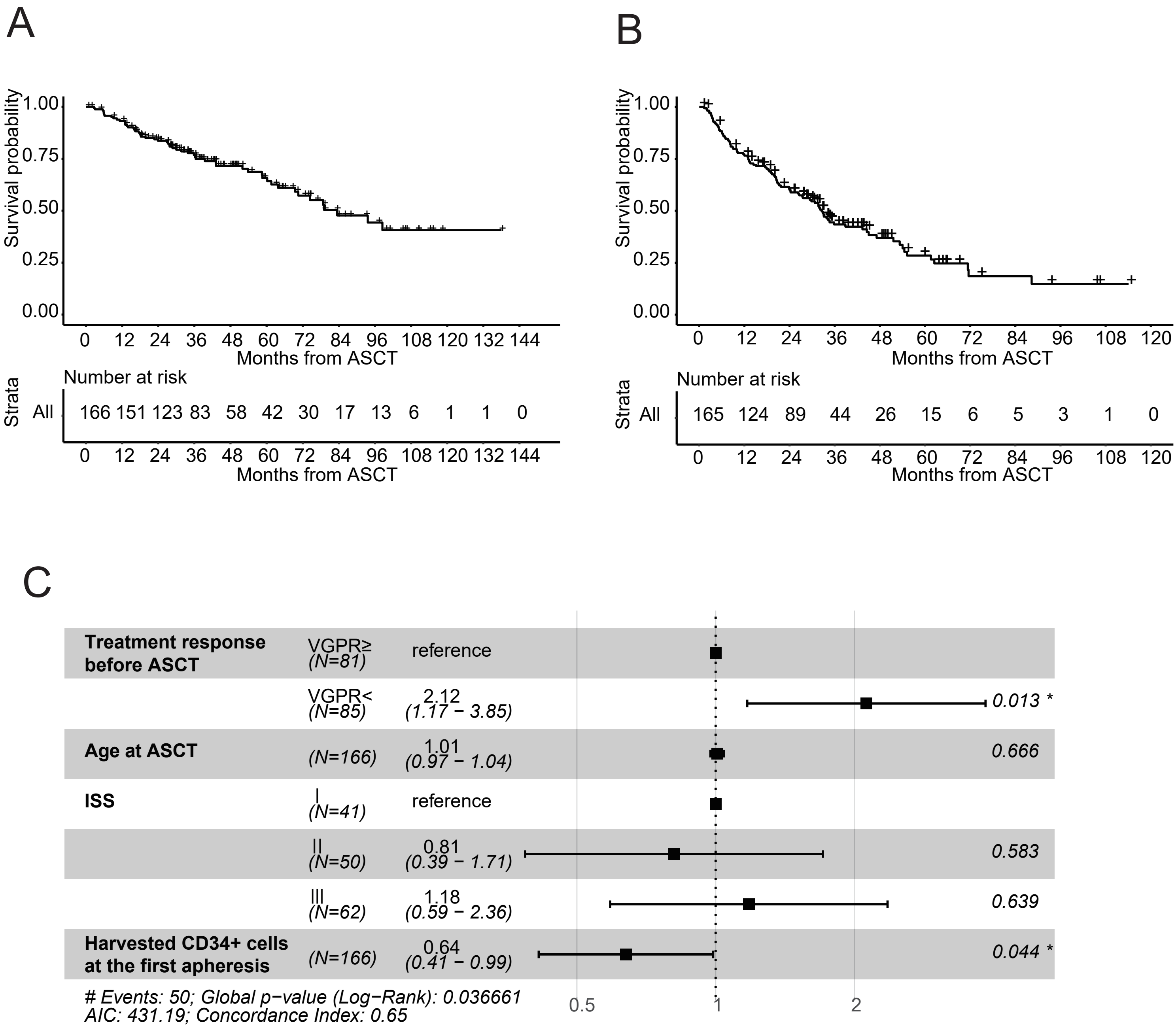

Supplemental Figure 2. Treatment effects before ASCT were independently associated with the OS of poor mobilizers who underwent ASCT.

(A) Overall survival from ASCT of poor mobilizers who underwent ASCT.
(B) Treatment-free survival from ASCT of poor mobilizers who underwent ASCT.
(C) A Cox proportional regression analysis of the overall survival of poor mobilizers who underwent ASCT.

Supplemental Table 1.

Initial treatment of poor mobilizers.

	Overall	With ASCT	Without ASCT
n	258	166	92
**Novel**	**202 (78.3)**	**131 (78.9)**	**71 (77.2)**
**PI+IMiDs**			
VRD/VRd-lite	24 (10.1)	22 (13.2)	4 (4.4)
KRd	2 (0.8)	0 (0.0)	2 (2.2)
VTD	1 (0.4)	0 (0.0)	1 (1.1)
**PI**			
Bd	106 (41.1)	66 (39.8)	40 (43.5)
Kd	1 (0.4)	0 (0.0)	1 (1.1)
VCD	56 (21.7)	38 (22.9)	18 (19.6)
VMP	2 (0.8)	2 (1.2)	0 (0.0)
VMD	1 (0.4)	0 (0.0)	1 (1.1)
PAd	1 (0.4)	0 (0.0)	1 (1.1)
**IMiDs**			
Rd	6 (2.3)	4 (2.4)	2 (2.2)
Td	2 (0.8)	1 (0.6)	1 (1.1)
**Chemotherapy**	**55 (21.3)**	**34 (20.5)**	**21 (22.8)**
VAD	34 (13.2)	20 (12.0)	14 (15.2)
MP	4 (1.6)	2 (1.2)	2 (2.2)
CAD	1 (0.4)	1 (0.6)	0 (0.0)
ROAD	1 (0.4)	0 (0.0)	1 (1.1)
HD-DEX	13 (5.0)	9 (5.4)	4 (4.3)
**RT**	**1 (0.4)**	**1 (0.6)**	**0 (0.0)**

Supplemental Table 2.

(A, B, C) Mobilization regimen of the first mobilization (A), second mobilization (B), and third mobilization (C).

A

	Overall	With ASCT	Without ASCT	p
n	258	166	92	
Number of apheresis (median [IQR])	2.00 [1.00, 2.00]	2.00 [1.00, 2.00]	2.00 [1.00, 2.00]	0.13
Mobilization regimen (%)				0.314
G-CSF	94 (36.4)	68 (41.0)	26 (28.3)	
CY	143 (55.4)	83 (50.0)	60 (65.2)	
CY+Bortezomib	8 (3.1)	6 (3.6)	2 (2.2)	
CY+VP-16	1 (0.4)	0 (0.0)	1 (1.1)	
VP-16	2 (0.8)	2 (1.2)	0 (0.0)	
DCEP	4 (1.6)	3 (1.8)	1 (1.1)	
VTD-PACE	3 (1.2)	2 (1.2)	1 (1.1)	
TD-PACE	1 (0.4)	1 (0.6)	0 (0.0)	
Unknown	2 (0.8)	1 (0.6)	1 (1.1)	
Use of plerixafor (%)	7 (2.7)	1 (0.6)	6 (6.5)	0.016

B

	Overall	With ASCT	Without ASCT	p
n	99	79	20	
Number of apheresis (median [IQR])	2.00 [1.00, 2.00]	2.00 [1.00, 2.00]	2.00 [0.75, 2.00]	0.72
Mobilization regimen (%)				0.722
G-CSF	37 (37.4)	30 (38.0)	7 (35.0)	
CY	39 (39.4)	29 (36.7)	10 (10.9)	
VP-16	19 (19.2)	16 (20.3)	3 (15.0)	
DCEP	3 (3.0)	3 (3.8)	0 (0.0)	
Unknown	1 (1.0)	1 (1.3)	0 (0.0)	
Use of plerixafor (%)	16 (16.2)	15 (19.0)	1 (5.0)	0.239
Harvested CD34+ cells (median [IQR])	1.45 [0.48, 4.55]	1.96 [0.96, 5.46]	0.23 [0.01, 0.43]	<0.001

C

	Overall	With ASCT	Without ASCT	p
n	10	8	2	
Number of apheresis (median [IQR])	2.00 [2.00, 2.75]	2.00 [2.00, 3.00]	2.00 [2.00, 2.00]	0.55
Mobilization regimen (%)				0.784
G-CSF	7 (70.0)	5 (62.5)	2 (100.0)	
CY	1 (10.0)	1 (12.5)	0 (0.0)	
VP-16	1 (10.0)	1 (12.5)	0 (0.0)	
Unknown	1 (10.0)	1 (12.5)	0 (0.0)	
Use of plerixafor (%)	6 (60.0)	5 (62.5)	1 (50.0)	1
Harvested CD34+ cells (median [IQR])	1.12 [0.78, 1.69]	1.32 [1.07, 2.14]	0.61 [0.56, 0.67]	0.068

Supplemental Table 3.

Baseline characteristics of poor mobilizers who underwent ASCT.

N	166
Age at ASCT (median [IQR])	62.55 [56.11, 65.99]
Infused CD34+ cells (×10^6/kg, median, range)	1.73 [0.70, 8.40]
Engraftment, Neutrophil (days, median [IQR])	12.00 [11.00, 13.00]
Engraftment, Platelet (days, median [IQR])	14.00 [12.00, 18.00]
Treatment response before ASCT (%)	
sCR	11 (6.6)
CR	8 (4.8)
VGPR	62 (37.3)
PR	77 (46.4)
SD	5 (3.0)
PD	3 (1.8)
Unknown	0 (0.0)
Treatment response after ASCT (%)	
sCR	18 (10.8)
CR	7 (4.2)
VGPR	68 (41.0)
PR	52 (31.3)
SD	4 (2.4)
PD	2 (1.2)
Unknown	15 (9.0)
Relapse (%)	97 (58.4)
Consolidation treatment (%)	51 (30.7)
Maintenance treatment (%)	63 (38.0)

Supplemental Table 4.

Treatment regimen of poor mobilizers after ASCT.
(A) Consolidation regimen of poor mobilizers after ASCT.
(B) Maintenance regimen of poor mobilizers after ASCT.

A

N	51
**PI+IMiDs**	
VRd	2
KRd	9
IRd	4
VTd	2
VPd	1
**PI**	
Bd	2
Kd	1
**IMiDs**	
R/Rd	18
T/Td	5
Pd	1
**Others**	
ERd	4
MP	1
CP	1

B

N	63
**PI**	
B/Bd	4
Ixazomib	3
**IMiDs**	
R/Rd	41
T/Td	8
**IMiDs+Ab**	
ERd	3
DRd	1
**Others**	
CP	1
Unknown	2

Supplemental Table 5.

Baseline characteristics according to the number of harvested CD34+ cells during the first mobilization.

N	CD34+cells<1.0×10^6/kg 135	CD34+cells≥1.0×10^6/kg 123	p
Age at diagnosis (median [IQR])	61.82 [57.08, 64.95]	60.96 [55.39, 64.77]	0.335
Age at apheresis (median [IQR])	62.47 [58.11, 65.75]	62.19 [56.03, 65.43]	0.324
Male sex (%)	70 (51.9)	59 (48.0)	0.618
Durie & Salmon stage (%)			0.399
I	10 (7.4)	15 (12.2)	
II	20 (14.8)	18 (14.6)	
III	99 (73.3)	81 (65.9)	
Unknown	6 (4.4)	9 (7.3)	
ISS stage (%)			0.523
I	33 (24.4)	37 (30.1)	
II	42 (31.1)	38 (30.9)	
III	54 (40.0)	40 (32.5)	
Unknown	6 (4.4)	8 (6.5)	
Cytogenetic abnormalities			
17p	12 (8.9)	6 (4.9)	0.308
t(4;14)	12 (8.9)	12 (9.8)	0.98
t(14;16)	4 (3.0)	2 (1.6)	0.766
Negative / Unknown	110 (81.5)	105 (85.4)	0.504
Number of treatment cycles before apheresis	4.00 [3.50, 6.00]	4.00 [4.00, 6.00]	0.724
Duration from diagnosis to apheresis (median [IQR])	5.83 [4.33, 8.93]	6.48 [4.70, 9.80]	0.349
Treated with lenalidomide-contained regimen (%)	42 (31.1)	47 (38.2)	0.286
Treatment response before apheresis (%)			0.427
VGPR≥	50 (37.0)	47 (38.2)	
VGPR<	79 (58.5)	74 (60.2)	
Unknown	6 (4.4)	2 (1.6)	
Mobilization regimen (%)			0.03
G-CSF+Chemotherapy	95 (70.4)	67 (54.5)	
G-CSF	39 (28.9)	55 (44.7)	
Unknown	1 (0.7)	1 (0.8)	
Harvested CD34+ cells at the first mobilization (median [IQR])	0.30 [0.02, 0.58]	1.56 [1.30, 1.77]	<0.001
Following ASCT (%)	62 (45.9)	104 (84.6)	<0.001

Supplemental Table 6.

Baseline characteristics of poor mobilizers who underwent ASCT according to the number of harvested CD34+ cells during the first mobilization.

	CD34+cells<1.0×10^6/kg	CD34+cells≥1.0×10^6/kg	p
n	62	104	
Age at ASCT (median [IQR])	62.56 [55.99, 66.36]	62.56 [56.13, 65.83]	0.9
Infused CD34+ cells (×10^6/kg, median, range)	1.92 [1.50, 3.18]	1.71 [1.48, 1.89]	0.02
Engraftment, Neutrophil (days, median [IQR])	12.00 [11.00, 13.00]	12.00 [11.00, 13.00]	0.68
Engraftment, Platelet (days, median [IQR])	16.00 [13.00, 20.50]	14.00 [12.00, 17.75]	0.02
Treatment response before apheresis (%)			0.366
VGPR≥	28 (45.2)	43 (41.3)	
VGPR<	33 (53.2)	61 (58.7)	
Unknown	1 (1.6)	0 (0.0)	
Treatment response before ASCT (%)			0.689
VGPR≥	32 (51.6)	49 (47.1)	
VGPR<	30 (48.4)	55 (52.9)	
Treatment response after ASCT (%)			0.607
VGPR≥	37 (59.7)	56 (53.8)	
VGPR<	21 (33.9)	37 (35.6)	
Unknown	4 (6.5)	11 (10.6)	
Consolidation treatment (%)	19 (30.6)	32 (30.8)	1
Maintenance treatment (%)	27 (43.5)	36 (34.6)	0.326
Relapse (%)	35 (56.5)	62 (59.6)	0.812

Supplemental Information

The following institutions (in alphabetical order) and investigators participated in this study.

Institution	Department	Investigator
Aiiku Hospital	Department of Internal Medicine and Hematology	Koh Izumiyama
Akita University Graduate School of Medicine	Department of Hematology, Nephrology, and Rheumatology	Sho Ikeda
Asahi General Hospital	Department of Hematology	Hiroaki Tanaka
Chiba Aoba Municipal Hospital	Department of Hematology	Katsuhiro Shono
Chiba Cancer Center	Division of Hematology-Oncology	Kyoya Kumagai
Chiba Rosai Hospital	Department of Hematology	Takeharu Kawaguchi
Chiba University Hospital	Department of Hematology	Emiko Sakaida
Chibaken Saiseikai Narashino Hospital	Department of Hematology	Ryuko Cho
Gunma University Graduate School of Medicine	Department of Medicine and Clinical Science	Hiroshi Handa
Hyogo Cancer Center	Department of Hematology	Tohru Murayama
Iwate Medical University School of Medicine	Division of Hematology & Oncology, Department of Internal Medicine	Shigeki Ito
Japan Community Health Care Organization Kyoto Kuramaguchi Medical Center	Department of Hematology	Shinichi Fuchida
Japanese Red Cross Medical Center	Division of Hematology	Nobuhiro Tsukada
Japanese Red Cross Narita Hospital	Department of Hematology and Oncology	Nobuyuki Aotsuka
Japanese Red Cross Wakayama Medical Center	Department of Hematology	Yutaka Shimazu
JCHO Funabashi Central Hospital	Department of Hematology	Motoharu Fukazawa
Juntendo University Urayasu Hospital	Division of Hematology	Yasunobu Sekiguchi
Kanagawa Cancer Center	Department of Oncology	Hiroyuki Takahashi
Kanazawa University	Department of Hematology	Hiroyuki Takamatsu
Keiju Kanazawa Hospital	Department of Internal Medicine	Takeshi Yamashita
Kyoto Prefectural University of Medicine	Division of Hematology and Oncology, Department of Medicine	Junya Kuroda
Miyagi Cancer Center	Department of Hematology	Yoriko Harazaki
Nagoya City University Graduate School of Medical Sciences	Department of Hematology and Oncology	Masaki Ri
Nagoya University Graduate School of Medicine	Department of Hematology and Oncology	Kazuyuki Shimada
National Hospital Organization Hiroshimanishi Medical Center	Department of Hematology	Yoshiaki Kuroda

National Hospital Organization Okayama Medical Center	Department of Hematology	Kazutaka Sunami
National Hospital Organization Shibukawa Medical Center	Department of Hematology	Morio Matsumoto
National Hospital Organization Tokyo Medical Center	Division of Hematology	Akihiro Yokoyama
National Hospital Organization Sendai Medical Center	Department of Hematology	Yuna Katsuoka
Nippon Medical School	Department of Hematology	Hideto Tamura
Oami Municipal Hospital	Department of Hematology	Koji Takaishi
Osaka Saiseikai Nakatsu Hospital	Department of Hematology	Teruhito Takakuwa
Sapporo City General Hospital	Department of Hematology	Satoshi Yamamoto
Sapporo Medical University School of Medicine	Department of Hematology	Hiroshi Ikeda
Shimane Prefectural Central Hospital	Department of Hematology and Oncology	Toshio Wakayama
Shimane University Hospital	Department of Oncology and Hematology	Takaaki Miyake
The Jikei University Kashiwa Hospital	Department Clinical Oncology and Hematology	Kazuhito Suzuki
The Jikei University School of Medicine	Department of Internal Medicine, Division of Clinical Oncology and Hematology	Kazuhito Suzuki
The University of Tokyo	Department of Hematology and Oncology, Graduate School of Medicine	Arika Shimura
Tokai University School of Medicine	Department of Hematology and Oncology, Department of Medicine	Rikio Suzuki
Tokushima Prefectural Central Hospital	Department of Hematology	Shuji Ozaki
Tokushima University Graduate School of Biomedical Sciences	Department of Hematology, Endocrinology and Metabolism	Shingen Nakamura
Toyama University Hospital	Department of Hematology	Akinori Wada
Wakayama Medical University Hospital	Department of Hematology/Oncology	Akinori Nishikawa
